# Effectiveness of heat and moisture exchangers in preventing ventilator-associated pneumonia in critically ill patients: a meta-analysis

**DOI:** 10.1186/1471-2253-14-115

**Published:** 2014-12-13

**Authors:** Mayra Gonçalves Menegueti, Maria Auxiliadora-Martins, Altacílio Aparecido Nunes

**Affiliations:** Escola de Enfermagem de Ribeirão Preto, Universidade de São Paulo, Ribeirão Preto, SP Brazil; Hospital das Clínicas da Faculdade de Medicina de Ribeirão Preto - USP, Divisão de Terapia Intensiva, Departamento de Cirurgia e Anatomia - 2o andar, Av. Bandeirantes, 3900 - Bairro Monte Alegre, Ribeirão Preto, SP Brazil; Departamento de Medicina Social, Av. Bandeirantes, 3900 - Bairro Monte Alegre, Ribeirão Preto, SP Brazil

**Keywords:** Ventilator-associated pneumonia, Heat and moisture exchangers, Critically ill patients

## Abstract

**Background:**

Patients may acquire ventilator-associated pneumonia (VAP) by aspirating the condensate that originates in the ventilator circuit upon use of a conventional humidifier. The bacteria that colonize the patients themselves can proliferate in the condensate and then return to the airways and lungs when the patient aspirates this contaminated material. Therefore, the use of HME might contribute to preventing pneumonia and lowering the VAP incidence. The aim of this study was to evaluate how the use of HME impacts the probability of VAP occurrence in critically ill patients.

**Methods:**

On the basis of the acronym "PICO" (Patient, Intervention, Comparison, Outcome), the question that guided this review was "Do critically ill patients under invasive mechanical ventilation present lower VAP incidence when they use HME as compared with HH?". Two of the authors of this review searched the databases PUBMED/Medline, The Cochrane Library, and Latin-American and Caribbean Literature in Health Sciences, LILACS independently; they used the following keywords: "heat and moisture exchanger", AND "heated humidifier", AND "ventilator-associated pneumonia prevention". This review included papers in the English language published from January 1990 to December 2012.

**Results:**

This review included ten studies. Comparison between the use of HME and HH did not reveal any differences in terms of VAP occurrence (OR = 0.998; 95% CI: 0.778–1.281). Together, the ten studies corresponded to a total sample of 1077 and 953 patients in the HME and HH groups, respectively; heterogeneity among the investigations was low (I^2^ < 50%). Information about the outcome mortality was available in only eight of the ten studies. The use of HME and HH did not afford different results in terms of mortality (OR = 1.09; 95% CI: 0.864–1.376). The total sample size was 884 and 762 patients, respectively. Heterogeneity among the studies was low (I^2^ = 0.0%).

**Conclusion:**

Current meta-analysis was not sufficient to definitely exclude an associate between heat and moisture exchangers and VAP. Despite the methodological limitations found in selected clinical trials, the current meta-analysis suggests that HME does not decrease VAP incidence or mortality in critically ill patients.

## Background

Nosocomial pneumonia remains to be one of the main causes of infection in intensive care units (ICU) [1, 2]. This condition is associated with the length of hospital stay [1, 3], duration of mechanical ventilation, and use of broad-spectrum antibiotics. In the particular case of Brazil, nosocomial pneumonia is the primary cause of infection among critically ill patients admitted to the ICU, which is associated with increased hospital costs [1, 2, 4–6] and mortality [3, 6]. This type of pneumonia occurs more frequently in patients submitted to mechanical ventilation for over 48 h, so it is commonly designated ventilator-associated pneumonia (VAP) [7]. VAP diagnosis relies on the emergence of a new pulmonary infiltrate or the presence of progressive pulmonary filtrate accompanied by fever, leukocytosis, and purulent secretion [7]. Mortality due to VAP varies between 24 and 50% and may reach rates as high as 76% in patients with comorbidities such as COPD, diabetes and other chronic lung diseases [8].

Mechanical ventilation suppresses the natural mechanisms that moisturize and heat inhaled air. When patients use an artificial airway, it is necessary to couple the ventilation system with a device that compensates for this suppression [9]. The lack of adequate moisturizing may thicken the secretions, which augments resistance to the passage of air, reduces the gas exchange effectiveness, and increases the risk of respiratory infections [10]. Air moisturizing and heating can be achieved actively (through the use of heated humidifiers, HH) or passively (by means of heat and moisture exchangers, HME) [1].

Patients may acquire VAP by aspirating the condensation of water (*ie*, overhumidification) that originates in the ventilator circuit upon use of a conventional humidifier. The bacteria that colonize the patients themselves can proliferate in the condensate and then return to the airways and lungs when the patient aspirates this contaminated material [10–12]. Therefore, the use of HME might contribute to preventing pneumonia and lowering the VAP incidence.

Many research papers have compared HME and HH in terms of VAP occurrence [13–17]. However, data about how effectively HME prevents VAP remain inconclusive. Hence, this study aimed to evaluate how the use of HME impacts the probability of VAP occurrence in critically ill patients.

## Methods

### Data sources and search strategies

This is a systematic review of the literature with meta-analysis. The methodology involved six stages: selection of the hypothesis, selection of the studies, definition of the characteristics, analysis of the studies included in this revision, interpretation of the results, and synthesis of the results.

On the basis of the acronym "PICO" (Patient, Intervention, Comparison, Outcome), the question that guided this review was "Do critically ill patients under invasive mechanical ventilation present lower VAP incidence when they use HME as compared with HH?". Two of the authors of this review searched the databases PUBMED/Medline, The Cochrane Library, and Latin-American and Caribbean Literature in Health Sciences, LILACS independently; they used the following keywords: "heat and moisture exchanger", AND "heated humidifier", AND "ventilator-associated pneumonia prevention". Table 1 presents the strategy these authors used to search for scientific evidence in the databases. This review included papers in the English language published from January 1990 to December 2012.

**Table 1:** **Search in electronic databases conducted on 01st December 2012**

Electronic base	Search strategy	Studies
**Medline**	(("Heat and Moisture Exchanger" AND ("heated humidifier") AND ("ventilator associated pneumonia" OR "ventilator associated pneumonia prevention"))	**1319**
**The Cochrane Library**	(("Heat and Moisture Exchanger" AND ("heated humidifier") AND ("ventilator associated pneumonia" OR "ventilator associated pneumonia prevention"))	**50**
	(Selection criteria: review studies and clinical assays)	
**LILACS**	(("Filtro Trocador de Calor e Umidade" E (Umidificador aquecido") ("Pneumonia associada à Ventilação Mecânica" OU "Prevenção de Pneumonia associada à Ventilação Mecânica")). (("Heat and Moisture Exchanger" AND ("heated humidifier") AND ("ventilator associated pneumonia" OR "ventilator associated pneumonia prevention"))	**93**

### Study selection

**Figure 1:**
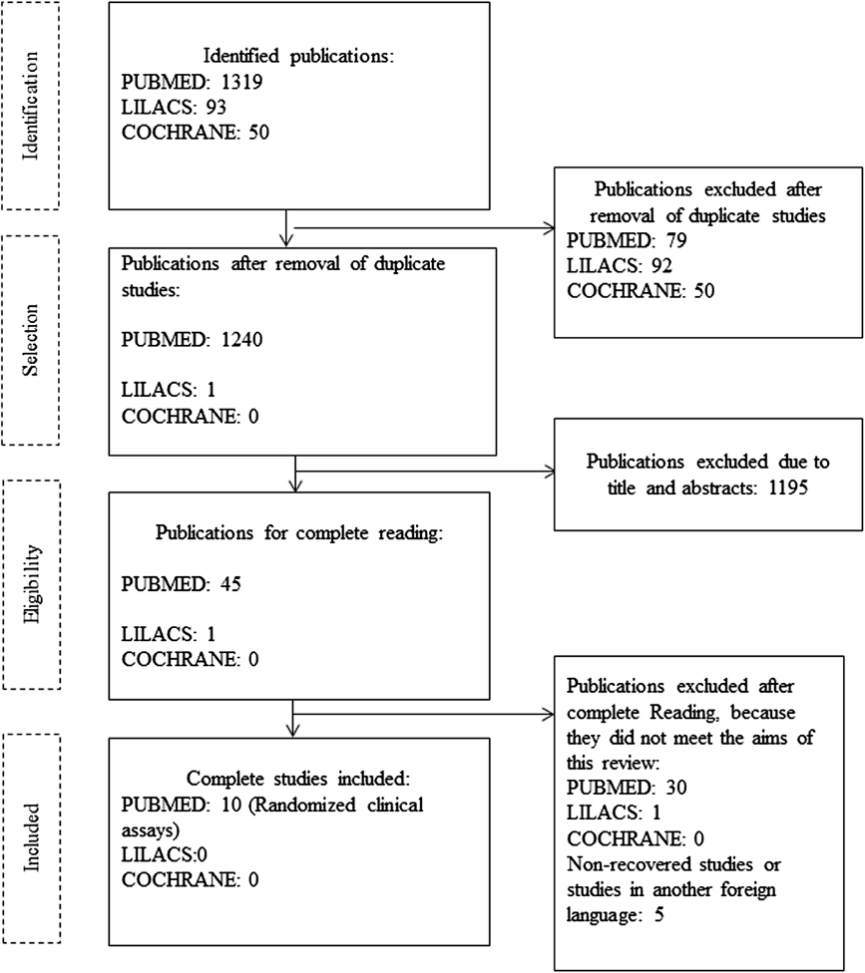
**Flow chart for the selection of studies included in this review.**

This review only considered controlled randomized clinical assays that evaluated the use of HME as compared with HH to prevent VAP in critically ill patients. Exclusion criteria included: studies that did not associate the use of exchanger with VAP, experimental (non-clinical) studies, economic assessments and reviews, and articles that were not fully available. The following information was relevant for data collection: (1) paper identification (paper and journal title, main author, year of publication, and study locations); (2) assessment criteria used in the studies (exchanger type); (3) methodological characteristics [study type, study aims, results (impact of the exchanger on VAP prevention and possible complications), limitations, and conclusions]. The final sample consisted of 10 articles. Figure 1 illustrates the inclusion process.

### Data analysis and statistical methods

The model to assess the quality of clinical assays proposed by the *Oxford Centre for Evidence-based Medicine–Levels of Evidence* (2009) helped to evaluate the quality of the evidence. Meta-analysis considered two outcomes, namely VAP occurrence and mortality rate among patients belonging to the HME (Intervention A) and the HH (Intervention B) groups, which meant that the outcomes were dichotomous. Odds Ratio (OR) and its 95% Confidence Interval (95% CI) were used for the results of each study and for the synthesis. The Cochran’s *Q* and the *I*-square (*I*^2^) tests aided evaluation of heterogeneity. All the data were reported considering 95% CI and two-tailed p values. The random effects model was employed in the case of significant heterogeneity among the studies (*I*^2^ > 50). The fixed effects model was applied for non-significant heterogeneity (*I*^2^ < 50). The meta-analysis was graphically represented by a Forest plot; the publication bias will be represented by a funnel plot. The software *Comprehensive Meta Analysis*™ version 2.2.064 (CMA Inc. USA) was employed to analyze and record data.

## Results

This review included ten studies. Table 2 summarizes the main results and depicts the characteristics of each publication in detail, including the level of evidence provided by each study.

**Table 2:** **Characterization of the studies on the use of heat and moisture exchangers as compared with the conventional humidifiers**

Study/Country	Setting	Data analysis	Study limitations	Complications	Other benefits	Evidence level
Martin et al., 1990 [23]	ICU	Quantitative variables were compared using the Student t test.	Pneumonia was diagnosed on the basis of purulent secretion. It did not involve VAP incidence density.	Hypothermia in 22% and 12% of the patients belonging to the HME and HH groups, respectively (p < 0.01). Six and no cases of tube occlusion were reported in the HME and HH groups, respectively (p < 0.01).	Not reported.	1C
				The study was interrupted after the death of a patient belonging to the HME group due to total obstruction of the endotracheal tube.		
Roustan et al., 1992 [26]	ICU	Both groups were compared using the Student t, Mann–Whitney, Chi-square, and Fisher exact tests for differences in frequency. Regression was conducted for the incidence of nosocomial pneumonia, atelectasis, and tube occlusion.	Sample size was not calculated.	Nine and no events of endotracheal tube occlusion in the HME and HH groups, respectively. Nine and ten episodes of atelectasis in the HH and HME groups, respectively.	Not reported.	1C
			The randomization procedure was not described.			
Dreyfuss et al., 1995 [22]	ICU	The Student t test was used for the continuous variables. The Chi-square test with Yates correction was employed for the categorical variables. The Mann–Whitney test was used to compare non-parametric variables.	The randomization procedure was not described. Various patients were excluded after randomization. Sample size calculation was not reported.	Report of severe occlusion that required cannula exchange due to clotting (patients with hematemesis) in the HME group. Six patients required cannula exchange due to obstruction by secretion in the HME group.	The use of HME reduces costs and staff working time.	1C
Boots et al., 1997 [21]	ICU	The patients’ characteristics were compared by paired t test. The VAP rate was evaluated using the log rank test.	The randomization procedure was not described.	Not reported.	The use of HME reduces costs.	1C
Kirton et al., 1997 [24]	ICU	Analysis of variance and non-paired Student t test.	Non-blinded study. ICU specifically admitted trauma patients.	The HME and HH groups did not differ in terms of endotracheal tube obstruction.	The use of HME reduces costs.	1C
Kollef et al., 1998 [25]	ICU	Student t and Wilcoxon tests were used (according to normal and non-normal distribution).	The randomization procedure was not described. No mention of blinded study.	Tube obstruction was not detected in any of the groups.	The use of HME reduces costs by 50%.	1C
		Chi-square and exact Fisher tests were employed to compare categorical variables.	Time elapsed during filter exchange was not controlled.			
		Results were confirmed by multiple logistic regressions.	VAP diagnosis criteria did not include bronchoalveolar lavage.			
Memish et al., 2001 [20]	ICU	The Student t test was employed.	The statistical power was not calculated.	Not reported.	Nursing staff spends less time discarding the condensate that builds up in the circuit. The use of HME reduces costs.	1C
Lacherade et al., 2005 [14]	ICU	The Student t test was employed for continuous variables. The Chi-square test was used for the categorical variables. Multivariate logistic regression was also performed.	Differences between the two populations with respect to HIV infection. Physicians and researchers were not blinded.	Tube occlusion rates were lower in the HME group (1 case) as compared with the HH group (5 cases).	On the basis of literature studies, the paper mentions that the use of HME reduces costs.	1C
Boots et al., 2006 [15]	ICU	Sample size was determined using the difference between two ratios. Univariate analysis involved the use of Student t and Kruskal-Wallis tests. The difference in VAP rate among groups was evaluated by Kaplan-Meier and log rank tests.	Pneumonia was diagnosed according to CPIS (Clinical Pulmonary Infection Score).	HME may present higher resistance to airflow than the manufacturer’s specifications after use for 24 h.	The use of HME reduces costs.	1C
Lorente et al., 2006 [19]	ICU	Quantitative variables were compared using the Student t test. Five risk models proportional to Cox were constructed for VAP analysis.	Temperature and moisture were not monitored. VAP diagnosis was confirmed by tracheal aspirate. After randomization, patients under mechanical ventilation for less than five days were excluded. Sample calculation was conducted, but it did not reach a sufficient number of patients. Wide confidence interval. Incidence density was not approached. Immunosuppressed patients were excluded.	Not reported.	No benefits have been reported for the use of HME.	1C

ICU: Intensive Care Unit; VAP: Ventilator-Associated Pneumonia; HME: Heat and moisture exchangers; HH: Heated Humidifiers.

**Figure 2:**
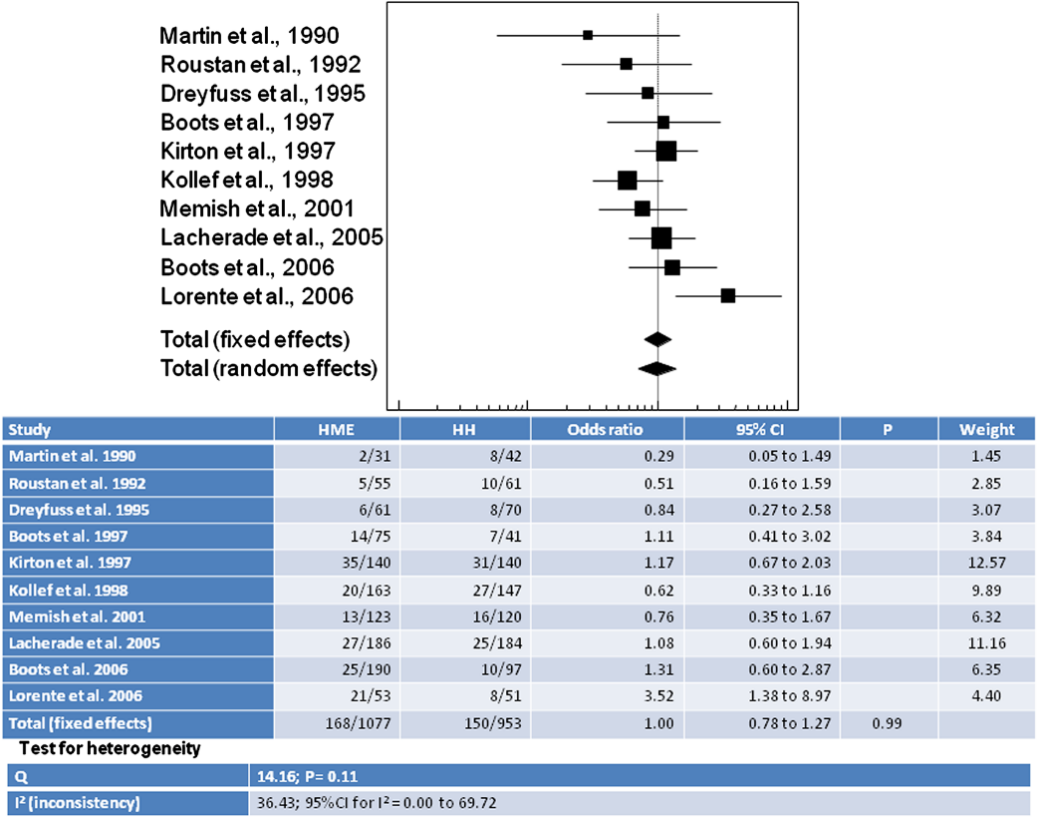
**Synthesis of the results considering the VAP outcome in the ten clinical assays selected for this review.**

Table 3 compares the VAP incidence and mortality in the different clinical assays.Meta-analysis provided a synthesis of the results considering the VAP outcome in the ten clinical assays selected for this review (Figure 2).

**Table 3:** **Description of the participants, VAP incidence, and mortality in the selected clinical assays**

Paper	Sample size		VAP incidence (% or per 1000 mechanically ventilated patients/day)		p value	Mortality (%)		p value
	HME group	HH group	HME group	HH group		HME group	HH group	
Martin et al., 1990 [23]	31 patients under MV	42 patients under MV	7%	19%	>0.05	22	26	>0.05
Roustan et al., 1992 [26]	55 patients under MV	61 patients under MV	9.1%	14.8%	>0.05	24.6	18.2	>0.05
Dreyfuss et al., 1995 [22]	61 patients under MV	70 patients under MV	9.8% (6 cases)	11.4% (8 cases)	>0.05	28	17	>0.05
			Confidence interval: 3.7-20.2%	Confidence interval: 5.1-21.3%				
Boots et al., 1997 [21]	75 patients under MV	41 patients under MV	19%	17%	>0.05	17.3	9.7	>0.05
Kirton et al., 1997 [24]	140 patients under MV	140 patients under MV	Early: 35 VAP cases per one thousand ventilated patients per day	Early: 31 VAP cases per one thousand ventilated patients per day	Early: p = >0.05	Data not shown		
					Late: < 0.05			
			Late: 12 VAP cases per one thousand ventilated patients per day	Late: 26 VAP cases per one thousand ventilated patients per day				
Kollef et al., 1998 [25]	163 patients under MV	147 patients under MV	20 VAP cases per one thousand ventilated patients per day	27.6 VAP cases per one thousand ventilated patients per day	Relative Risk: 0.90	24.5	26.5	>0.05
					CI: 0.46-1.78			
					p >0.05			
Memish et al., 2001 [20]	123 patients under MV	120 patients under MV	13.3 VAP cases per one thousand ventilated patients per day.	15.7 VAP cases per one thousand ventilated patients per day.	p >0.05	28.8	25	> 0.05
Lacherade et al., 2005 [14]	186 patients under MV	184 patients under MV	27.4 VAP cases per one thousand ventilated patients per day	25.3 VAP cases per one thousand ventilated patients per day	0.76	32.4	34.2	> 0.05
					Odds Ratio (0.58–1.57)			
					p > 0,05			
Boots et al., 2006 [15]	190 patients	2 HH groups	13%	- Group with heating upon inspiration: 10%	0.61	15	- Group with heating upon inspiration: 14	> 0.05
		- Group with heating upon inspiration: 94						
		- Group with heating upon inspiration and expiration: 97		- Group with heating upon inspiration and expiration: 10%			- Group with heating upon inspiration and expiration: 22	
Lorente et al., 2006 [19]	53 patients under MV	51 patients under MV	21 episodes (39%)	8 episodes (15%)	Hazard Ratio 16.2 (4.54-58.04)	Data not shown		
					p < 0.05			

HME: Heat and moisture exchangers; HH: Heated Humidifiers; MV: Mechanical Ventilation; VAP: Ventilator-Associated Pneumonia.

**Figure 3:**
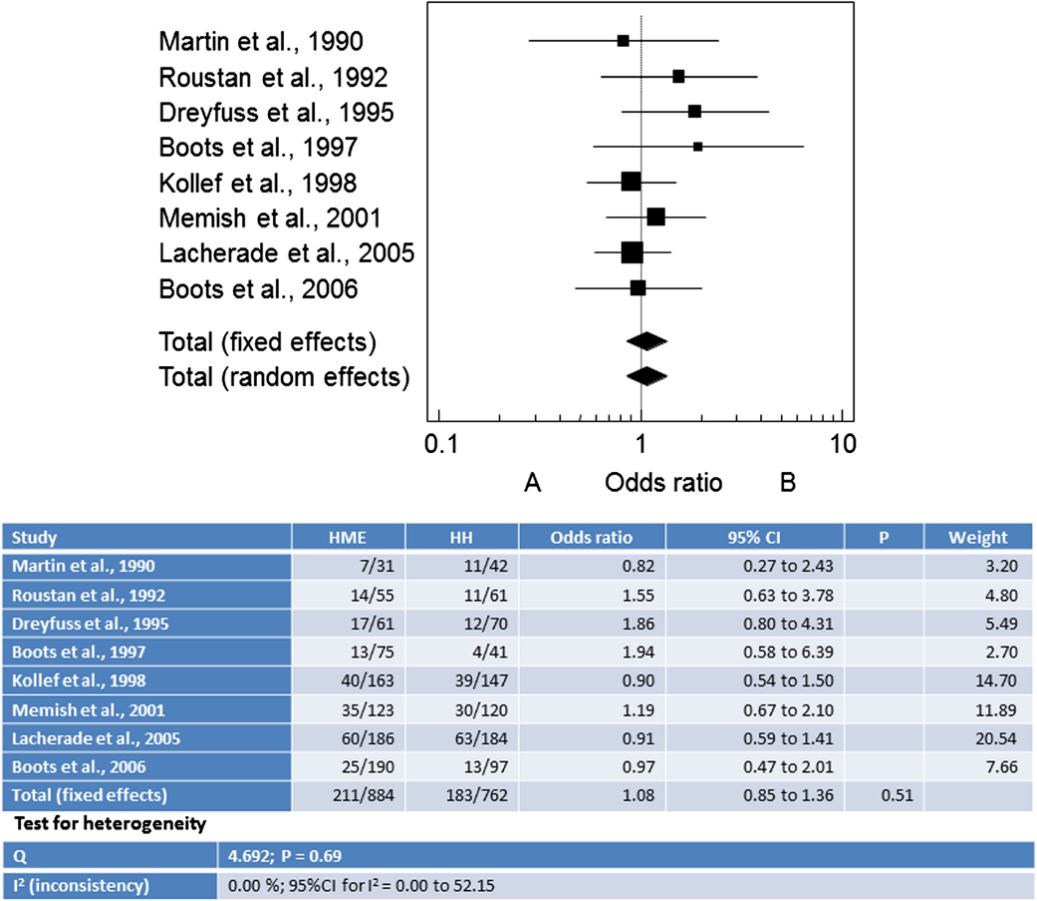
**Synthesis of the results considering mortality in eight of the ten studies.** Meta-analysis containing Forest plot and Odds Ratio values of each study and summarized Odds Ratio by the fixed effects model with their respective 95% CI; beyond the value of Q and I^2^ tests, considering HME **(A)** and HH **(B)**.

Comparison between the use of HME and HH, in this meta-analysis did not reveal any differences in terms of VAP occurrence (OR = 0.998; 95% CI: 0.778–1.281). Together, the ten studies corresponded to a total sample of 1077 and 953 patients in the HME and HH groups, respectively; heterogeneity among the investigations was low (I^2^ < 50%).Information about the outcome mortality was available in only eight of the ten studies. Hence, the results synthesis can be seen in the referent meta-analysis (Figure 3).

**Figure 4:**
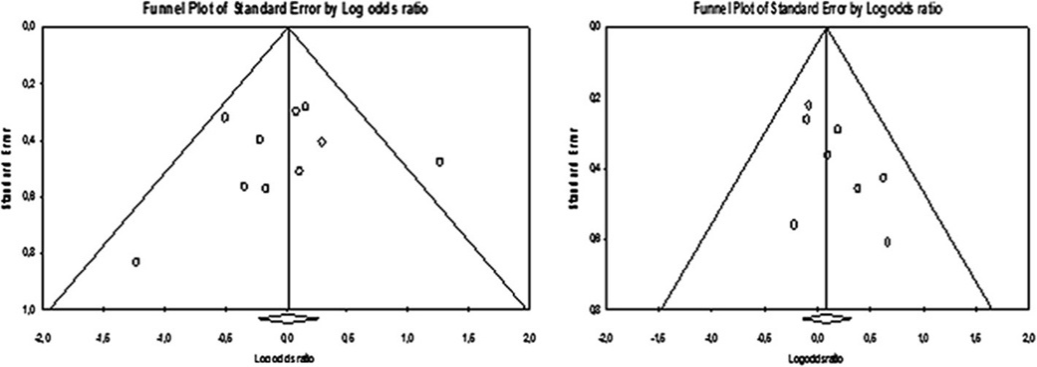
**Considering the two outcomes investigated here, there was no evidence any publication bias.** Funnel plot showing that there is no evidence of publication bias on VAP **(A)** and mortality **(B)** outcomes.

In this meta-analysis the use of HME and HH was not associated with different rates/risk of mortality (OR = 1.09; 95% CI: 0.864–1.376). The total sample size was 884 and 762 patients, respectively. Heterogeneity among the studies was low (I^2^ = 0.0%).Considering the two outcomes investigated here, Figure 4 does not evidence any publication bias.

## Discussion

Literature studies have presented conflicting results about how HME impacts VAP prevention. A meta-analysis including nine studies and 1368 patients revealed that HME reduced VAP rates especially in the case of subjects submitted to mechanical ventilation for over seven days (RR = 0.7; IC 95%: 0.50-0.94). However, non-randomized studies (not included in this meta-analysis) found significantly lower VAP rates in the groups that used HH as compared with HME [18]. Other two randomized studies reported non-significantly different VAP rates for HH and HME [14, 15]. A randomized study of 120 patients demonstrated smaller VAP incidence during the use of HH in patients under mechanical ventilation for periods longer than five days (15.69 vs 39.62%, P = 0.006) [19]. A meta-analysis published by Siempos et al. (2007) [16] included 13 randomized clinical assays and 2,580 patients; it did not detect any differences between HME and HH with respect to VAP incidence, mortality in the ICU, length of stay in the ICU, period of mechanical ventilation, or airway obstruction.

Most studies have found similar VAP rates for HME and HH [14–18, 20–26]. In most of these studies, the HME and HH groups did not differ in terms of VAP incidence density, period of mechanical ventilation, length of stay in the ICU, or global mortality rate.

Kirton et al. (1997) [24] evaluated 280 trauma patients and demonstrated lower VAP incidence during the use of HME. On the other hand, Auxiliadora-Martins et al. (2012) [17] investigated severely ill patients (mean APACHE II score > 25) with associated comorbidities, to find that the HH and HME group did not have different VAP incidence. The heterogeneous study populations, the distinct brands of HME employed in the aforementioned studies, the frequency with which the nursing staff changed the moisturizer, and the criteria used to diagnose VAP may have contributed to these contrasting results [19]. Nevertheless, HME did offer advantages over HH: it did not require that the staff opened the device circuit to remove the condensate accumulated in the ventilator extensions, which reduced the occupational risk and optimized the work of the nursing team. In addition, it may have some disadvantages using HH; such as high maintenance costs and mechanical malfunction of the humidification and mechanical ventilator apparatus, and overheating of the inspired gases. These problems could be avoided by using HME [10–12, 17].

Even though many studies have shown that the use of HME incurs lower costs [14, 15, 21, 22, 24, 25], this device is not risk-free. Partial or total tube occlusion is the most probable adverse effect, and its frequency is significantly different in patients submitted to HME and HH [14, 22, 23, 26]. Lower moisture production may account for this difference, especially during application of larger minute volumes. This is a common event in all the commercially available devices, albeit to different extent [27–29]. Martin et al. (1990) [23] have also described more frequent cases of hypothermia in patients using HME. In contrast, Kirton et al. (1997) [24] and Kollef et al. (1998) [25] did not observe any differences between the HH and HME groups in terms of tube occlusion. The use of HME involves other concerns: increased dead volume due to hypercapnia and larger airway resistance, which depend mainly on the filter internal volume and liquid accumulation, respectively [29–31].

In most studies the change of HME was every 24 or 48 hours according manufacturer’s recommendation. Other studies have demonstrated that prolonged use of HME, from 24–48 h up to four or seven days, does not incur increased risk of VAP [31–35]. Davis et al. did not detect reduced efficiency, increased resistance, or altered bacterial colonization when the use of HME was discontinued after three days. The VAP incidence rate did not change, either, so the authors concluded that the use of HME for over 24 h, up to 72 h, is safe and economically advantageous.

Together, the analyzed studies suggest that it is possible to use either HH or HME without significantly impacting the VAP incidence. Considering hospital costs and in the absence of contraindications, HME should be employed as an alternative humidifier in patients submitted to mechanical ventilation. The results of the present review agree with the pathophysiology proposed for VAP in the sense that bacteria inoculation into the lungs generally occurs via extraluminal source, whereas only occasionally does the intraluminal pathway happen.

Considering a 30% reduction in the occurrence of VAP among patients who used respectively, HH (16%) and HME (11%), with a significance level of 5% and a statistical power of 80%, the minimum sample per group would be 731 subjects. In the present meta-analysis, it was evaluated studies with 953 subjects in the HH group and 1077 in the HME group, thus this review presents a sufficient sample size to detect this difference. However there are some potential limitations should be considered. Firstly, the sample size was sufficient to detect a 30% reduction in VAP rates; the power of the study was not sufficient to identify more modest reductions, although smaller reductions could still be important from a clinical perspective. Secondly, some studies were not calculated sample size and were not described the randomization procedure. Thirdly, in two studies patients were excluded after randomization and other two studies were non blinded. Finally, the definitions of VAP varied in some included studies. Thus other double-blind studies, randomized controlled should be performed to definitely exclude an associate between heat and moisture exchangers and VAP.

Hence, the studies included in this meta-analysis did not attest that the use of HME in the ICU setting reduced the VAP incidence or affected mortality rates. On the basis of these two outcomes (VAP incidence and mortality), the meta-analysis of the selected studies was reliable, given that the samples had low heterogeneity.

## Conclusion

Current meta-analysis was not sufficient to definitely exclude an associate between heat and moisture exchangers and VAP. Despite the methodological limitations found in selected clinical trials, the current meta-analysis suggests, with some degree of uncertainty, that HME does not decrease VAP incidence or mortality in critically ill patients and therefore, further clinical trials with greater methodological rigor and adequate sample size should be performed comparing HH to HME in the incidence of VAP and other important outcomes such as mortality. Is important to highlight that, institutions routinely using HME should be aware of obstruction events and cases of hypercapnia and hypothermia, because some studies have described their occurrence.

### Consent

Written informed consent was obtained from the patient for the publication of this report.
